# Functional improvement of patients with Parkinson syndromes using a rehabilitation training software

**DOI:** 10.3389/fneur.2023.1210926

**Published:** 2023-08-14

**Authors:** Marcus Barth, Robert Möbius, Peter Themann, Erdem Güresir, Cornelia Matzke, Dirk Winkler, Ronny Grunert

**Affiliations:** ^1^Department of Neurosurgery, Faculty of Medicine, Leipzig University, Leipzig, Germany; ^2^Clinic at Tharandter Forest, Department of Neurology and Parkinson, Halsbruecke, Germany; ^3^Department of Medical Engineering, Fraunhofer-Institute for Machine Tools and Forming Technology, Dresden, Germany

**Keywords:** Exergame, Parkinson’s disease, rehabilitation, Kinect, physiotherapy, home-based, markerless, movement training

## Abstract

**Introduction:**

Individuals with Parkinsonian disorders often face limited access to specialized physiotherapy and movement training due to staff shortages and increasing disease incidence, resulting in a rapid decline in mobility and feelings of despair. Addressing these challenges requires allocating adequate resources and implementing specialized training programs to ensure comprehensive care and support. Regarding these problems, a computer software was invented that might serve as an additional home-based extension to conventional physiotherapy.

**Methods:**

The trial took place in a rehabilitation center where every patient received equivalent treatment apart from the training program that was set up to be investigated over 3 weeks. Seventy four Patients were included and randomized between two intervention and one control group. Intervention group 1 (IG1) trained with the computer-based system two times a week while Intervention group 2 (IG2) received five training sessions a week. Using the markerless Microsoft Kinect® camera, participants controlled a digital avatar with their own body movements. UPDRS-III and Clinical measurements were performed before and after the three-week period.

**Results:**

Patients in all groups improved in UPDRS-III pre and post intervention whereas reduction rates were higher for IG1 (−10.89%) and IG2 (−14.04%) than for CG (−7.74%). Differences between the groups were not significant (value of ps CG/IG1 0.225, CG/IG2 0.347). Growth rates for the arm abduction angle were significantly higher in IG1 (11.6%) and IG2 (9.97%) than in CG (1.87%) (value of ps CG/IG1 0.006 and CG/IG2 0.018), as was the 5-steps-distance (CG 10.86% vs. IG1 24.5% vs. UG2 26.22%, value of ps CG/IG1 0.011 and CG/IG2 0.031).

**Discussion:**

The study shows the beneficial effects of computer-based training and substantiates the assumption of a similar impact in a home-based setting. The utilized software is feasible for such interventions and meets with the patient’s approval. Group dynamics seem to have an additional supporting effect for the aspired objective of improving mobility and should be seen as an essential aspect of video games in therapy.

## Introduction

The treatment of patients with Parkinson’s disease and other Parkinsonian disorders necessitates a comprehensive and multidimensional approach that incorporates medication, physiotherapy, ergotherapy, psychotherapy, and social assistance (1). Empowering patients with autonomy and self-determination in their battle against the disease, along with the pursuit of therapy effectiveness, serves as the driving force behind the development of an independent exercise therapy tailored to this specific group of patients.

Previous reviews have demonstrated the feasibility and mostly comparable effects of video games in the treatment and rehabilitation of individuals with various neurological disorders (2–4). However, the current availability and design of these gamified experiences primarily cater to healthy users, typically children or young adults. Consequently, individuals facing individual limitations due to disorders, mobility restrictions, and age often find themselves excluded from participating in these activities or utilizing them for medical treatment purposes. To address this issue, our group, comprising clinical doctors, rehabilitation physicians, and software engineers, undertook the endeavor of creating a camera-assisted exercise medium that allows this specific patient group to compensate for physical deficits associated with the disease within the comfort of their own homes.

Exergames, also known as exercise games, have demonstrated significant utility in the treatment of patients with Parkinsonian disorders (5). In line with this, a special virtual reality training game utilizing the Microsoft Kinect® camera was developed in collaboration with an experienced software company. This innovative approach combines the benefits of exergaming and virtual reality technology to provide a tailored and engaging exercise experience for individuals with Parkinsonian disorders. Accordingly, Canning et al. (6) highlight the increasing demand for virtual reality technology in rehabilitation settings and the need for further research in this area.

Building upon a previously conducted pilot study (7), this clinical trial was conducted to investigate the benefits of the system within a cohort of patients with Parkinsonian disorders, taking into account the scarcity of controlled studies on the subject (8) and the insufficient training dosage (9) or sample size (10) in previous research. Furthermore, this study aimed to specifically examine the hypothesis that a higher frequency of additional computer-based training would result in greater improvement in mobility and movement among patients. Additionally, research has shown that training with video games such as Kinect®-based exercises can enhance cognitive aspects (11, 12), which offers the prospect of similar benefits in the domains of cognition and motivation.

Moreover, this approach represents a potential response to the increasing incidence of Parkinson’s disease resulting from demographic changes, as well as the shortage of physiotherapists (13). By establishing a home-based treatment model, one-on-one care becomes less necessary. Additionally, it is widely acknowledged that physical exercise through exergaming can improve both quality of life and balance in patients with PD (14). Besides, specific phenomena such as Pisa syndrome in PD (15) and freezing of gait (16) can and should be addressed through diverse exercises. Furthermore, these innovative applications of telemedicine can help reduce costs associated with travel and therapy itself (17). Although these challenges are not new, they have gained significant attention, particularly in light of the coronavirus pandemic.

## Methods

### Design

#### Inclusion, exclusion, and attrition

**Figure 1:**
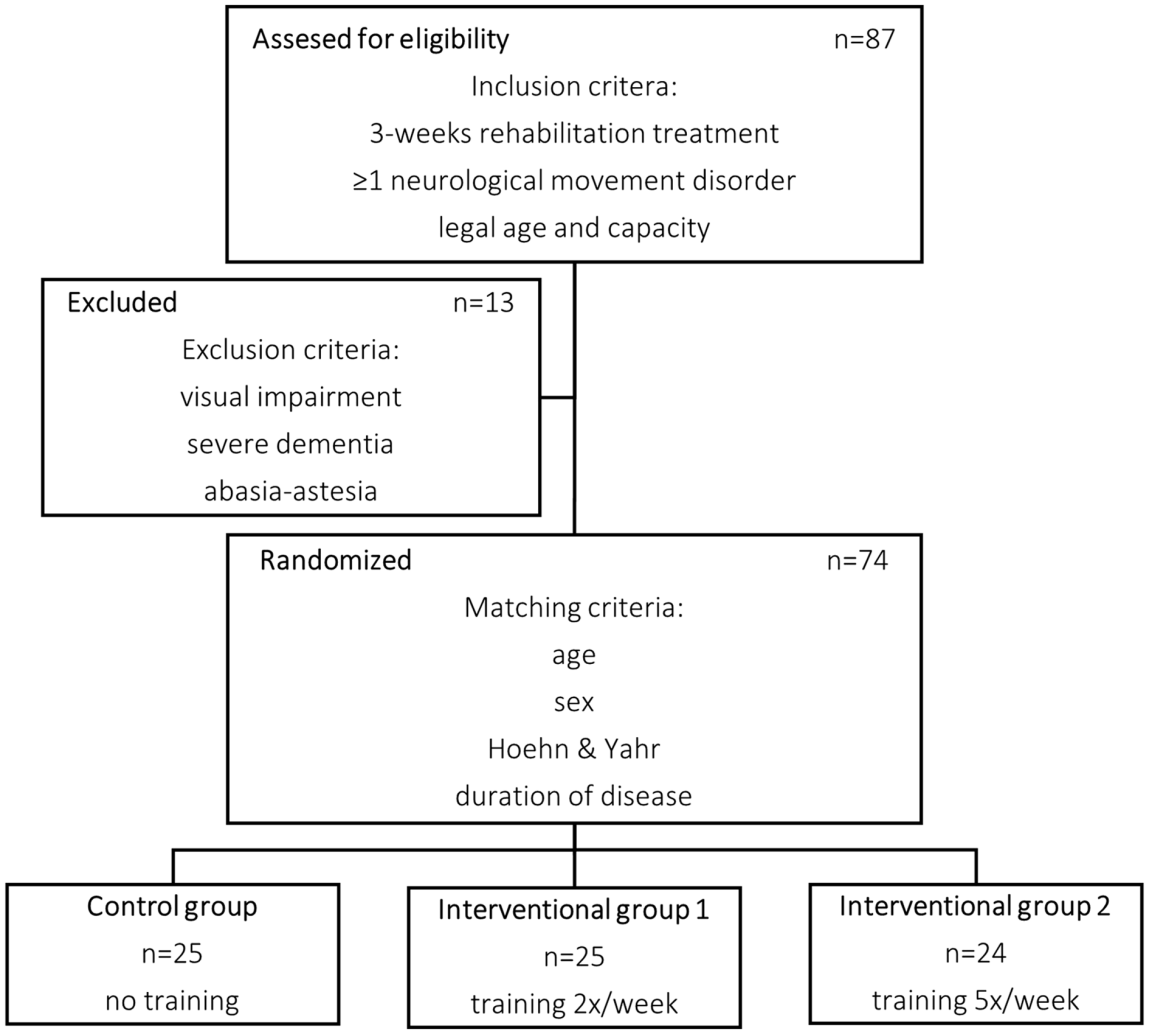
Flow chart of patient selection.

The trial was designed following a prospective, randomized, and controlled protocol. The investigation was conducted at a rehabilitation hospital focused on neurology patients and certified as a rehabilitation center for Parkinsonian diseases. Primarily, 87 potential patients were identified of which 74 were eventually included (Figure 1). Patients were included if they had a rehabilitation treatment of at least 3 weeks, had a diagnosis of at least one neurological movement disorder, were of legal age, and had capacity. Exclusion criteria were severe visual impairments, severe dementia, and inability to walk. During the course of the study, there was one instance of attrition where a patient was unable to complete the full 3-week protocol. This occurred because the patient experienced recurring syncopes and orthostatic instability unrelated to this trial, requiring an acute referral to another hospital for further medical intervention. As a result, the data from this particular case had to be excluded from the analysis to maintain the integrity and consistency. By removing the incomplete data from the analysis, the overall validity of the study’s findings can be preserved.

#### Patient selection

The selected patients were randomized using a matched pairs design dividing them into three groups: Intervention group 1 and Intervention Group 2 (IG1 and IG2) containing 25 and 24 patients, respectively, and a control group (CG) containing 25 patients. To ensure balanced groups and minimize potential bias, a merging process was implemented to assign patients to triplets based on their baseline characteristics at a rough estimate. Within each triplet patients were then randomly assigned to one of the three treatment groups helping to distribute any potential confounding factors equally among the groups and enhancing the validity of results. Baseline characteristics including age, number of patients with DBS system, Hoehn and Yahr score, duration of disease, and duration of rehabilitation treatment were similar between each group (Table 1). There was no further group stratification based on DBS.

**Table 1:** Baseline data.

	Control group (Group 1, no training)	Interventional group 1 (Group 2, training 2x/week)	Interventional group 2 (Group 3, training 5x/week)	*p*-value
No. of participants	25 [m 16, f 9]	25 [m 15, f 10]	24 [m 15, f 9]	
Age (years, mean)	72.92 (± 9.65)	73.56 (± 9.28)	72.71 (± 8.00)	0.570
No. of participants with DBS	3 (12%)	3 (12%)	4 (17%)	0.860
Hoehn and Yahr score (mode)	3 [1–4]	3 [1–4]	3 [1–4]	0.760
Duration of disease (years, mean)	8.64 (± 6.00)	8.32 (± 8.03)	7.83 (± 5.87)	0.785
Duration of rehabilitation treatment (weeks, width)	3 [3–6]	3 [3–4]	3 [3–5]	0.785
Levodopa equivalent dose pre (mg, mean)	603.44 (± 331.64)	582.08 (± 393.71)	652.58 (± 369.91)	0.716
Levodopa equivalent dose post (mg, mean)	641.52 (± 360.18)	649.12 (± 371.85)	719.58 (± 377.07)	0.701
Levodopa dose pre (mg, mean)	394.00 (± 223.17)	339.00 (± 239.91)	373.96 (± 250.60)	0.551
Levodopa dose post (mg, mean)	389.00 (± 207.43)	364.00 (± 246.34)	389.67 (± 245.15)	0.839

The purpose of the study, the associated risks, potential outcomes, and the anonymized usage of data were thoroughly explained to the patients who were assigned. The informed consent process was conducted, and the patients provided their consent in written form, indicating their understanding and agreement to participate. It was also made clear that they had the right to refuse participation or withdraw from the trial at any point without the need to provide reasons. By providing comprehensive information and obtaining informed consent, the study adhered to ethical guidelines and ensured that the patients were well-informed participants in the research process.

#### Training scheme

An idealized training scheme was set up to distribute training days balanced throughout the total intervention time of 3 weeks. Meanwhile, all patients continued receiving standard rehabilitation physical therapy and medical optimization in terms of medication and non-medication assistance. Patients in the IG1 trained twice weekly, either on Mondays and Thursdays or on Tuesdays and Fridays resulting in a total of six training days. Patients in the IG2 trained every day within the week (Mondays to Fridays), thereby receiving 15 days of exercise in total. Patients in the CG were treated with conventional rehabilitation therapy only.

#### L-Dopa equivalents

To exclude the adaption of medication as a disruptive factor for the interpretation of changes in the physical agility of patients, L-Dopa medication was assessed pre- and post-intervention. L-Dopa equivalent doses were calculated to ensure comparability. Following the introduction of safinamide in 2015 and opicapone in 2016, the previously used conversion (18) was extended (19) and utilized in this trial. Table 1 shows that all three groups increased their L-Dopa equivalent dose whereas there is no difference between each of them in both pre- and post-assessment. It was not feasible to conduct a more precise registration of On–off-Status and exact medication administration per day and per patient. Therefore, these specific details were not recorded or included in the study’s data collection process.

### Training system

#### Development

The inventory process and the intended purpose of this system were arranged in concordance with the latest suggestions by the MDS Task Force on Technology (20). While inventing the training system, primary body movement disorders such as gait and balance disorders, camptocormic posture and gait abnormalities, rigidity, akinesia, tremor, and fine motor skills disorders were identified, most of which were integrated into the conceptual planning. Specific movement patterns were defined which are to be practiced with the support of the therapy system and which counteract the above-mentioned disorders in a targeted manner. Particular attention was paid to stretching the upper body and getting the patient to stand up and sit down addressing greater walking and standing stability and the speed of movement. Another requirement for the system was the recognition of essential symptoms via marker-free sensor systems. Despite its approved usage for diagnostical purposes (21, 22), it was not to be assumed at the current time of processing, that resting, action and postural tremors of the hands could be recorded with the Microsoft® Kinect sensor system. To avoid fatigue and other adverse reactions, the duration of the training had to be adjusted accordingly and break times were considered. Cognitive exercises are integrated into the system to bridge them.

#### Specifications

**Figure 2:**
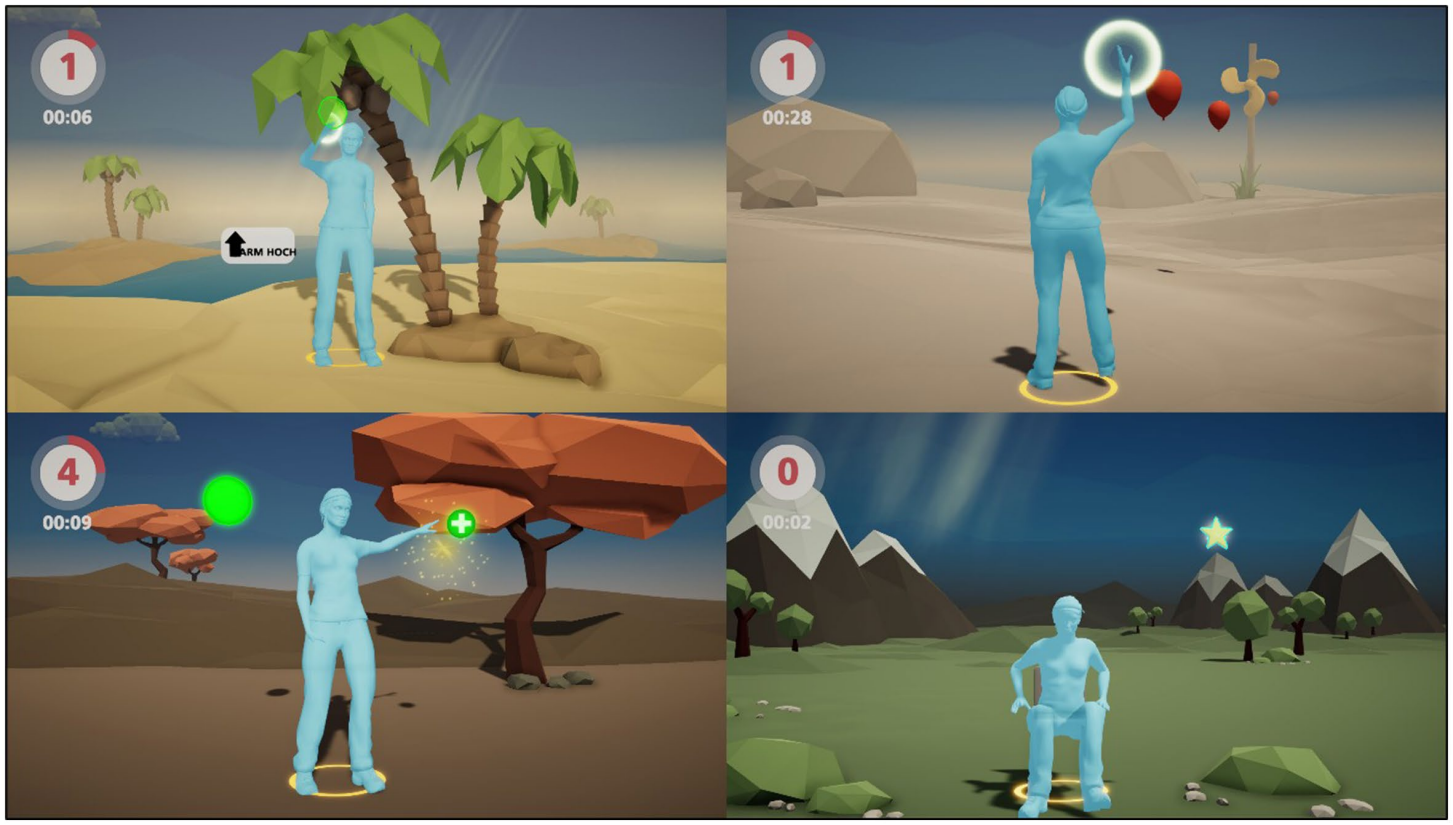
Game situation for the Coconuts, Balloons, Balls and Stars Games (from top left to bottom right).

The established training system is based on the markerless sensing Microsoft Kinect® camera which can easily be connected to any computer system and runs with the developed training software without additional software needed. Moreover, the setup requires a simple LCD monitor such as a TV set that most people possess in their homes. For the training session, the patient has to stand in front of the camera at a distance of two to four meters and needs enough space to move their arms freely. No additional software or hardware is needed which makes the system very feasible and safe to use. The software includes guiding instructions for the whole game, as well as previews for all the movements the patients have to perform within the different games making it completely self-explanatory. Symptoms of Parkinsonian diseases tend to progress with time showing a successive decline in movement amplitude and speed (23). To counteract these developments the games were designed to condition a faster and wider sequence of movement. Similar concepts of progressive training have been used by Vieira de Moraes Filho et al. (24) eventually improving brady- and akinesia over time. In an overall game time of approximately 20 min, patients play four mini-games (Figure 2) with a focus on upper limb movement and the rise from a chair targeting the extension of the range and speed of motion. Table 2, therefore, comprises the intended purposes for every specific movement trained by one of the four exercises. Additionally, the software records the height of the avatar and the time needed to trigger each following object within the different games to assess the progress of the player afterwards.

**Table 2:** Description of games and addressed movements.

Name of exercise	Movement trained	Purpose
Coconuts	Upper limbs, abduction/elevation	Reach out to objects above the head
Stars	Lower limbs, hip and knee extension	Stand up from a chair
Balls	Upper limbs/upper body, abduction/retroversion and rotation	Reach out to objects behind the shoulder, stabilize the body
Balloons	Upper limbs, anteversion	Reach out to objects in front

### Data collection

#### Measurements

Clinical examinations and measurements included in this study were made by the conducting staff and by well-trained physiotherapists. To objectify the findings, the change in the Unified Parkinson’s Disease Rating Scale (part III) of The International Parkinson and Movement Disorders Society (MDS-UPDRS-III) was used as the primary outcome variable. This scale is well established for the assessment of motoric symptoms of patients with Parkinsonian diseases (25). The data collection was conducted following an idealized schedule with an observation period of 3 weeks (21 days) for every patient. Clinical measurements and MDS-UPDRS-III were taken before the first and after the last training session of the interventional groups and on the first and last day of the three-week study term of the CG, respectively. The assessments mentioned are routine procedures conducted during admissions to the rehabilitation center where the trial took place. To ensure the accuracy and consistency of the assessments, the team of physiotherapists responsible for conducting the UPDRS-III assessments received comprehensive training from experienced neurologists. As a result, they were highly proficient in administering the tests and were well-versed in the evaluation process. The UPDRS-III assessments were performed on an individual basis and in a single-blinded manner. This means that the conducting physiotherapists were unaware of both the training status and group assignment of each patient. This approach helped minimize potential bias and ensured the objectivity of the assessments.

To extend the evaluation to record elusive changes in mobility that are not covered by the findings of the MDS-UPDRS-III assessment, further clinical measurements and tests were applied. The patients were asked to stand straight and stretch out their arms as far above their heads as possible. Then, the distance from the fingertips to the floor was measured to investigate the maximal erecting of the body. This is meant to address the ability to reach out to objects that are placed overhead which is a very important skill in day-to-day life. Apart from that, the greatest abduction angle of both arms was documented by taking photographs and was later quantified using the graphical software GIMP®. The same software was used to evaluate the angle of camptocormia as a marker for the severity of the posture impairment. Additionally, patients had to do a 5-step-walking test which primarily focused on freezing symptoms after the initial “start” command and which was meant to assess gait impairments. Therefore, time and distance were recorded. Due to the nature of the study and the involvement of the main researcher in both the training sessions and data collection, maintaining blinding for data acquisition of these additional measurements was not feasible. However, efforts were made to ensure objectivity and consistency in the data collection process.

#### Statistics

Statistical analysis was conducted using IBM SPSS Statistics 25.0® (Armonk, United States). The normal distribution of the outcome parameters was assessed using the Shapiro–Wilk test, given the small sample size. Depending on the nature of the data, either parametric or non-parametric test procedures were employed. Specifically, the Mann–Whitney U-test and Kruskal-Wallis test were used for continuous variables, while the Pearson’s Chi^2^ test was applied for categorical variables. A significance level of 0.05 was used for all test procedures.

## Results

All groups showed improvements regarding MDS-UPDRS-III and clinical measurements within the observed period.

### MDS-UPDRS-III

**Figure 3:**
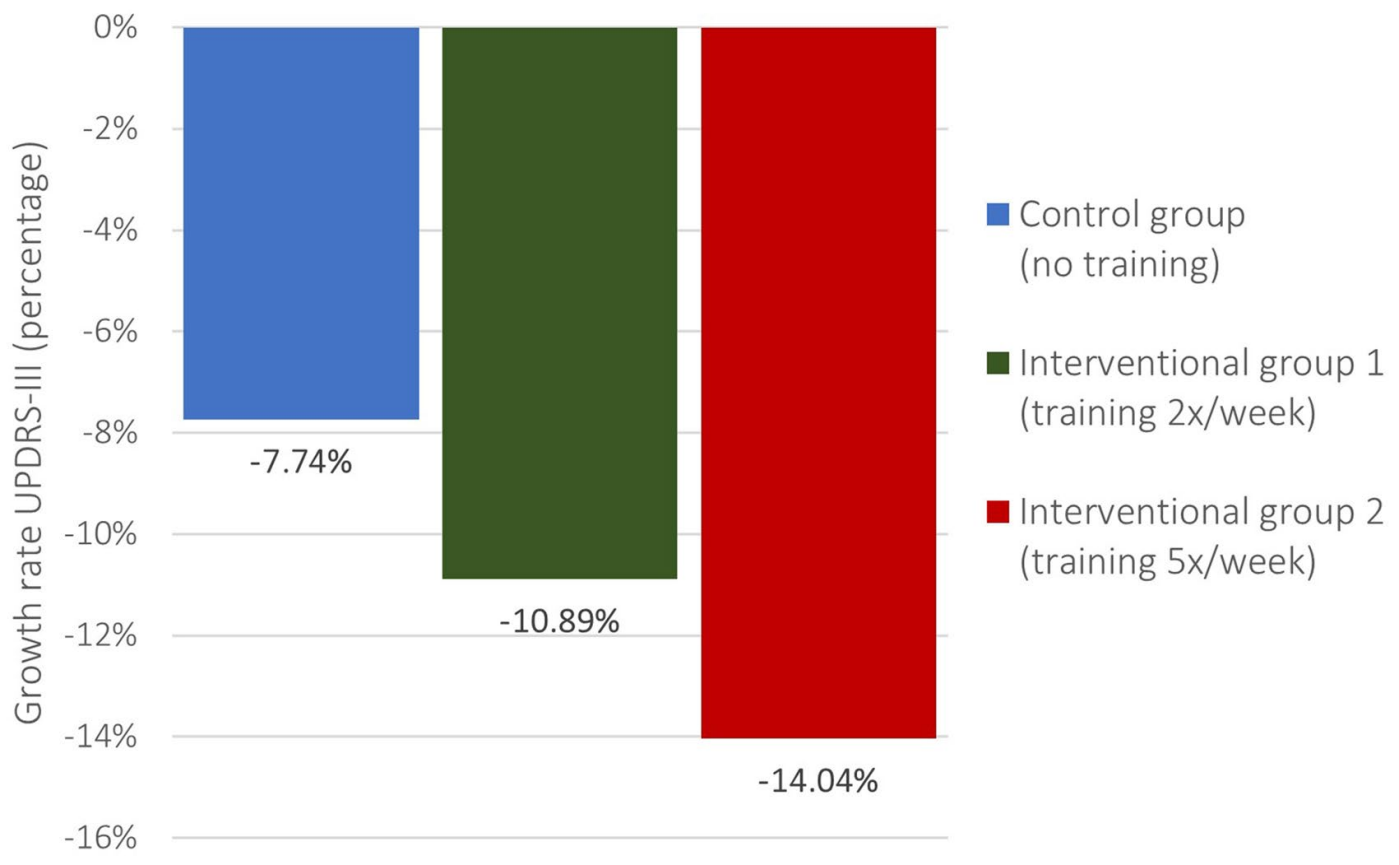
MDS-UPDRS-III growth rates.

**Figure 4:**
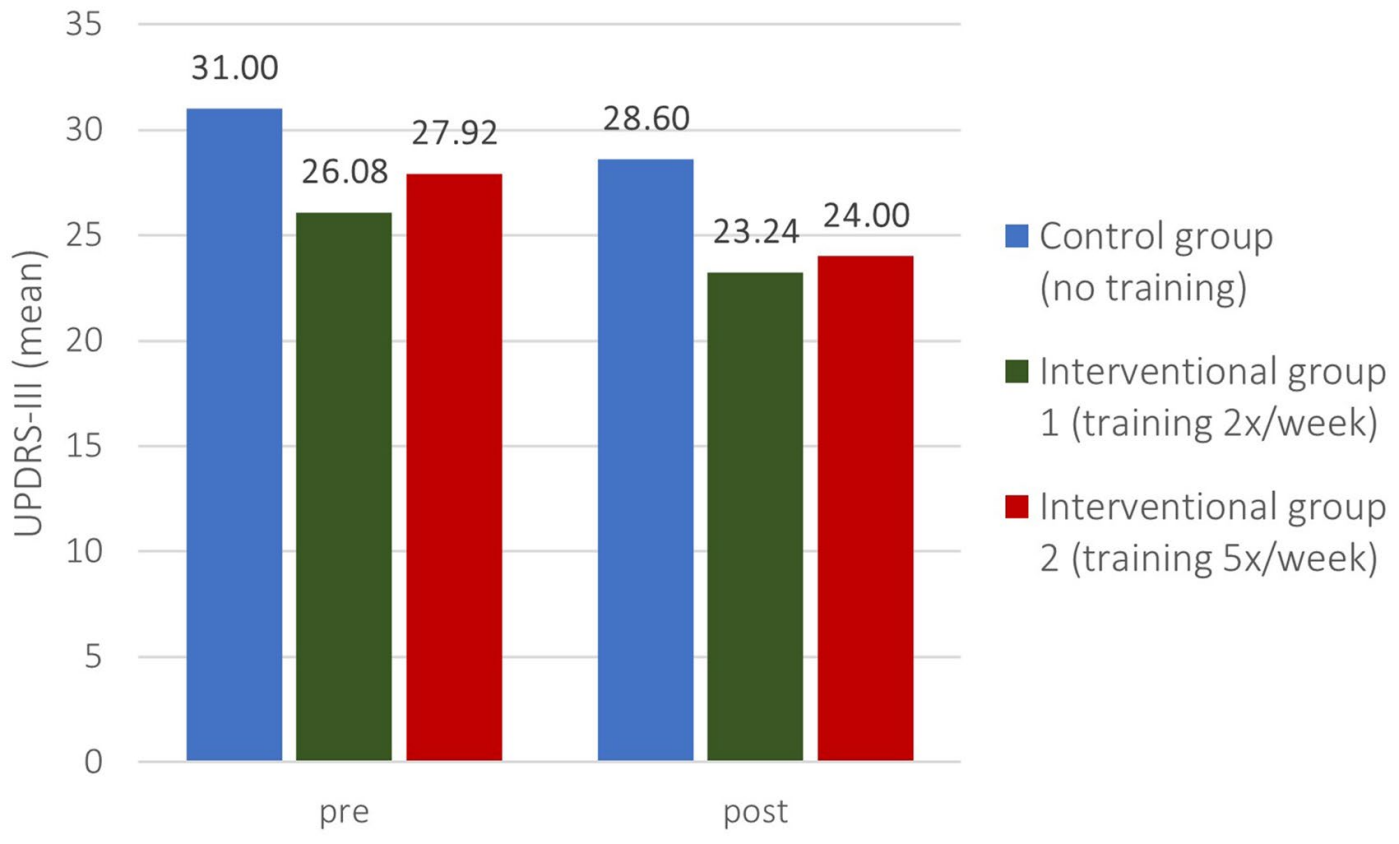
MDS-UPDRS-III pre- and post-intervention.

Patients presented with insignificantly different MDS-UPDRS-III at baseline (value of ps CG/IG1 0.214, CG/IG2 0.418) between the two Interventional Groups (26.08 IG1, 27.92 IG2) and the Control Group (31.0 CG). Improvements in motion result in lower MDS-UPDRS-III which is why calculated growth rates are negative. The reduction rate (Figure 3) in the IG1 (−10.89%) was higher and in the IG2 (−14.04%) nearly double as it was in the CG (−7.74%) which correlates with an absolute reduction (Figure 4) of −2.84 (IG1), −3.92 (IG2) and − 2.4 (CG), respectively (Table 3). Nevertheless, these findings were not significant (value of ps CG/IG1 0.225, CG/IG2 0.347).

**Table 3:** MDS-UPDRS-III pre- and post-intervention, MDS-UPDRS-III growth rate.

	Control group (Group 1, no training)	Interventional group 1 (Group 2, training 2x/week)	Interventional group 2 (Group 3, training 5x/week)	Value of p (Group 1 vs. group 2)	Value of p (Group 1 vs. group 3)
UPDRS III pre (mean)	31.00 (± 16.51)	26.08 (± 14.82)	27.92 (± 14.56)	0.214	0.418
UPDRS III post (mean)	28.60 (± 17.66)	23.24 (± 15.47)	24.00 (± 14.43)	0.225	0.347
Growth rate	−7.74%	−10.89%	−14.04%		

### Clinical measurements

**Figure 5:**
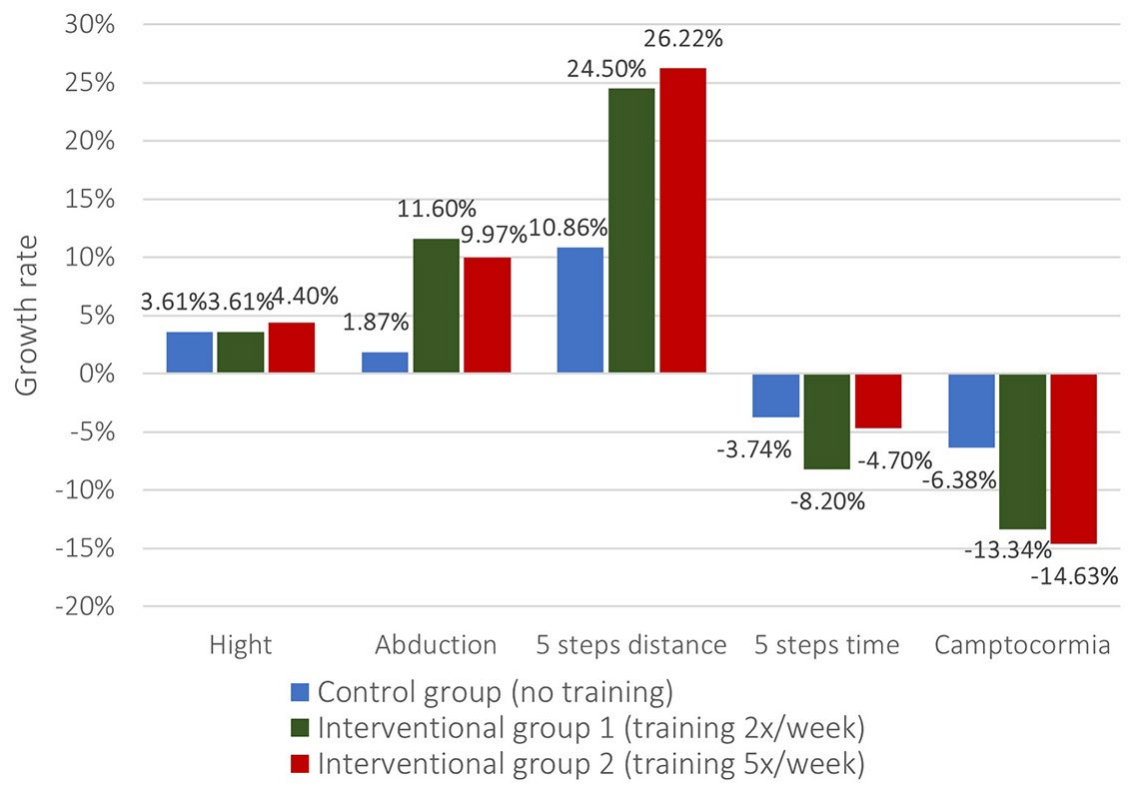
Growth rates of changes in clinical measurements.

Accordingly, the clinical measurements present similar findings (Figure 5). While there could not be identified significant differences in height and 5-step-time, the interventional groups differed significantly from the control group in terms of abduction angle and 5 steps distance (Table 4). For the abduction angles the growth rate in the CG was only 1.87% compared to 11.60% in the IG1 and 9.97% in the IG2 (value of p CG/IG1 0.006, CG/IG2 0.018). Likewise, growth rates in the 5-step-distance doubled with 24.50% in the IG1 and 26.22% in the IG2 compared with 10.86% in the CG (value of p CG/IG1 0.011, CG/IG2 0.031). Further, the improvement of camptocormia was better in the interventional groups as well (−13.34% IG1 / -14.63% IG2 vs. -6.38% CG) but lacking in significance.

**Table 4:** Growth rates of changes in clinical measurement.

(Growth rates, mean)	Control group *(Group 1, no training)*	Interventional group 1 *(Group 2, training 2x/week)*	Interventional group 2 *(Group 3, training 5x/week)*	*p*-value *(Group 1 vs. group 2)*	*p*-value *(Group 1 vs. group 3)*
Hight	3.61	3.61	4.4	0.236	0.133
Abduction	1.87	11.6	9.97	**0.006**	**0.018**
5 steps distance	10.86	24.5	26.22	**0.011**	**0.031**
5 steps time	−3.74	−8.2	−4.7	0.377	0.984
Camptocormia	−6.38	−13.34	−14.63	0.648	0.244

Significant findings are highlighted in bold.

### Kinect® data

**Figure 6:**
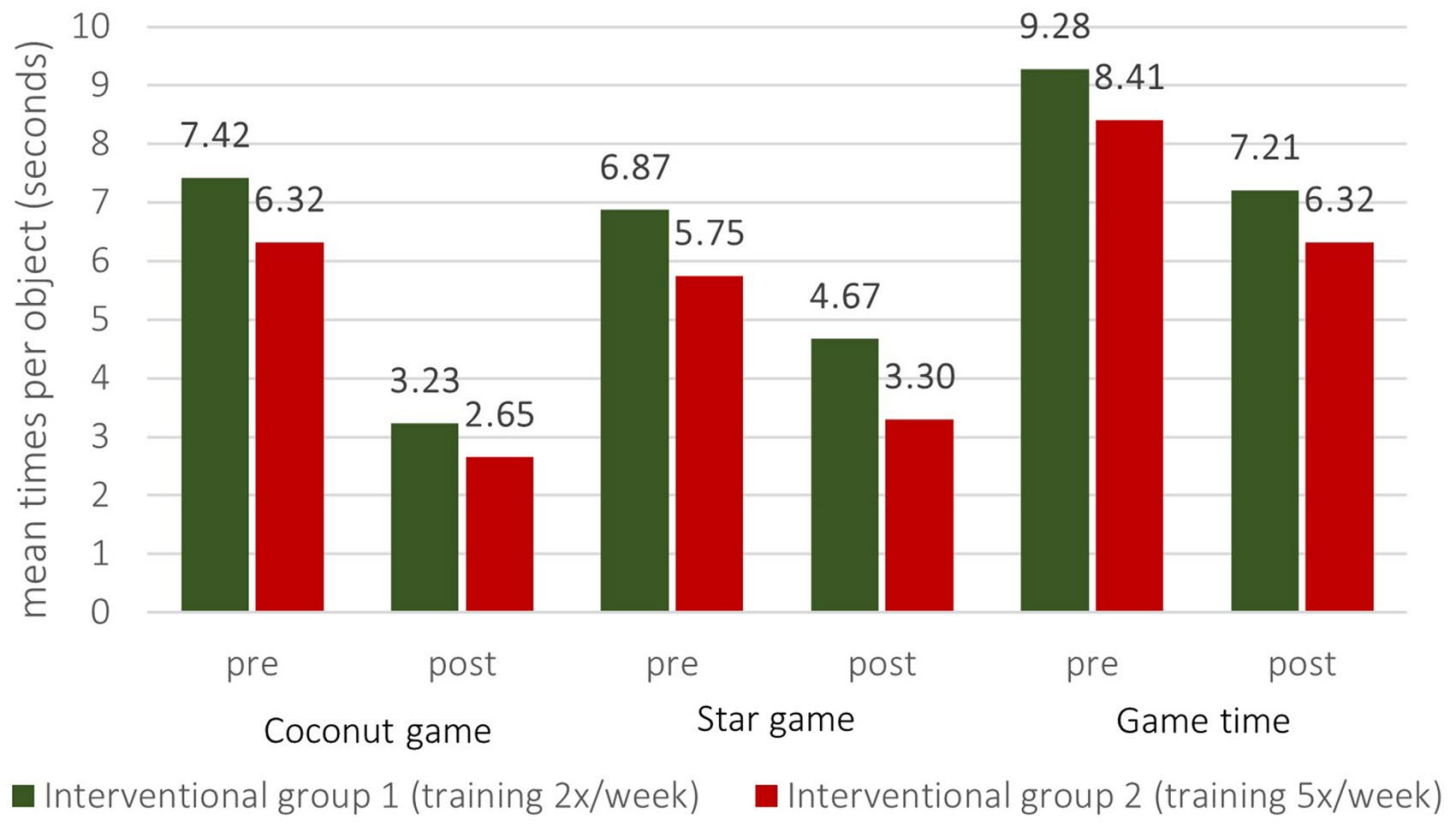
Means of time to trigger subsequent objects pre- and post- intervention per group.

As previously mentioned, the Microsoft Kinect® camera can track and record parameters within the game. That made it possible to assess the influence of the training through the system itself accordingly and to compare these findings to the other results as a further system evaluation. As the intervention was only performed on the IG1 and IG2, differences between these two groups were analyzed (Figure 6). At baseline, there were no significant differences between the groups regarding the means of the duration needed to trigger subsequent objects. On the last day of the training, patients in the IG2 became significantly faster in the “Coconut game” and in the “Overall game time” than in the IG1, whereas similar improvements seen in the “Star game” were tightly insignificant (Table 5).

**Table 5:** Means of time to trigger subsequent objects pre- and post-intervention per group.

	(Means)	Interventional group 1 (Group 2, training 2x/week)	Interventional group 2 (Group 3, training 5x/week)	*p*-value
Coconut game	Pre	7.42	6.32	0.575	Post	3.23	2.65	**0.036**
Star game	Pre	6.87	5.75	0.447	Post	4.67	3.30	0.063
Game time	Pre	9.28	8.41	0.327	Post	7.21	6.32	**0.013**

Significant findings are highlighted in bold.

## Discussion

### Outcome

The results of this study provide strong evidence supporting the positive impact of rehabilitation treatment on the movement abilities of patients with Parkinsonian diseases. These findings further support previous research indicating the beneficial effect of inpatient rehabilitation settings (26). All groups exhibited improvements in the assessed attributes over the observed period, indicating the effectiveness of various aspects of the treatment, including medication, physiotherapy, sociopsychological dynamics, and Kinect®-based training.

### MDS-UPDRS-III and clinical examination evaluation

Combining conventional rehabilitation treatment with computer-based training sessions demonstrated greater advancements in movement abilities. While the statistical significance of improvements in MDS-UPDRS-III and clinical examinations varied, it can be assumed that the use of the MS Kinect®-based training system can enhance mobility for patients with Parkinson’s disease. Specifically, the training system had a positive effect on the movement range of the upper limbs, step length, and posture of the patients. Higher frequency of additional training correlated with greater improvements, with the interventional groups outperforming the control group and Intervention Group 2 showing even better results than Intervention Group 1. Weaker improvements in abduction degree and 5-steps-time in IG2, compared to IG1, can be attributed to some patients in IG2 undergoing joint replacement procedures, which limited their limb mobility independent of Parkinson’s disease symptoms.

### Kinect® data evaluation

The Kinect® system records were found to be valid and consistent with other findings. The fact that IG2 demonstrated increasingly faster completion of game quests compared to IG1 supports the assumption of a direct positive correlation between mobility improvement and training frequency. These results underscore the feasibility, safety, and benefits of using the Kinect® system to assist physiotherapy. However, further enhancements to the software are necessary to tailor it to individual disease levels and enhance patient motivation for daily training sessions.

### Group dynamics

One noteworthy aspect of training games, as observed in this study, is the positive impact of group dynamics reported by the staff. This factor may have influenced the beneficial effects of the game therapy on the patients and warrants further investigation. The enjoyment derived from this novel form of rehabilitation training positively impacted motivation for each subsequent session, aligning with findings from previous studies (27–29). Additionally, considering the theoretical prevention of Parkinson’s disease (30), moderate physical exercise should be recommended to younger, healthy individuals due to the epidemiological correlation between higher physical activity and lower incidence of the disease (31).

### Conditioning

Another contributing factor to the observed improvements was patient conditioning through repeated use of the same training games. Patients derived satisfaction and motivation from noticing their performance improvements, fostering a sense of pride in their achievements. This, in turn, contributed to the group dynamics mentioned earlier and a heightened drive for better results in each subsequent training session. Similarly, a study by Schootemeijer et al. (32) reported significantly higher adherence to exercise in highly motivated patients. This suggests that motivated individuals are more likely to maintain long-term exercise habits, leading to consolidated effects and sustained benefits. These observations may also be attributed to improvements in working memory and cognitive function (33) as well as enhancements in functional connectivity between the cortex and basal ganglia (34).

### Limitations

Several limitations affected the consistency of statistical significance, including the relatively small sample size and adjustments in medication during the study period. Changes in L-Dopa administration, in particular, could have introduced some inequality. However, the lack of significance in the slight differences observed between the groups indicates that serious deviations were unlikely. Nevertheless, the increased L-Dopa doses in each group probably had a proportional effect on mobility improvement, highlighting the importance of optimizing medication in the treatment of patients with Parkinsonian diseases. Furthermore, the limited number of patients with deep brain stimulation (DBS) hampers the analysis of any potential effects of DBS, which will be explored in future research. Studies with a similar 3-week design have shown consistent findings, and long-term maintenance of exercises for several years has demonstrated a relative stabilization of impairing symptoms (35). This suggests that continuous engagement in exercise therapy can have prolonged benefits. Additionally, due to the three-week observation period, this study was unable to assess positive long-term effects on Parkinson’s disease progression, as observed in other therapeutic studies (36, 37). Indeed, research with a comparable design but longer follow-up periods has demonstrated significant effects as early as 12 weeks (38), providing further support for the presumed efficacy of the presented intervention.

### Summary

In summary, this study demonstrates the beneficial effects of computer-based training and supports the assumption that similar impacts can be achieved in a home-based setting. The software and hardware used in the intervention were feasible and well-received by the patients. Group dynamics emerged as an essential aspect of video game therapy, offering additional support for the goal of improving mobility.

## Data availability statement

The raw data supporting the conclusions of this article will be made available by the authors, without undue reservation.

## Ethics statement

The studies involving human participants were reviewed and approved by Ethikkommission der Westsächsischen Hochschule Zwickau. The patients/participants provided their written informed consent to participate in this study.

## Author contributions

MB handled the clinical implementation of the training system, conducted the training sessions with patients, gathered the data, wrote the article and created the tables and figures. RM provided technical expertise with the training system, helped in organizing the data collection, and conducted the data analysis. PT was involved in the conceptual planning of the training system, provided facilities for patient enrolment, gave clinical advice throughout the trial conduction, and critically revised the article. EG was involved in the conceptual planning of the training system and its clinical implementation. CM was involved in the conceptual planning of the training system and its clinical implementation. DW was involved in the conceptual planning of the training system, determined the study design, edited the article after critical revision and organized the trial conduction. RG co-created the training system, was involved in its conceptual planning and clinical implementation, provided technical expertise and critically revised the article. All authors contributed to the article and approved the submitted version.

## Funding

This work was supported by the Federal Ministry for Economic Affairs and Climate Action BMWK, “Central Innovation Programme for small and medium-sized enterprises” with the grant number 16KN051636. The authors acknowledge support from the German Research Foundation (DFG) and Universität Leipzig within the program of Open Access Publishing.

## Conflict of interest

The authors declare that the research was conducted in the absence of any commercial or financial relationships that could be construed as a potential conflict of interest.

## Publisher’s note

All claims expressed in this article are solely those of the authors and do not necessarily represent those of their affiliated organizations, or those of the publisher, the editors and the reviewers. Any product that may be evaluated in this article, or claim that may be made by its manufacturer, is not guaranteed or endorsed by the publisher.

## Abbreviations

CG: Control Group
DBS: Deep Brain Stimulation
IBM: International Business Machines Corporation
IG1: Intervention Group 1
IG2: Intervention Group 2
LCD: Liquid-crystal display
L-Dopa: Levodopa
MDS: The International Parkinson and Movement Disorder Society
PD: Parkinson’s disease
TV: Television
UPDRS: Unified Parkinson’s Disease Rating Scale
